# HES5-mediated repression of LIGHT transcription may contribute to apoptosis in hepatocytes

**DOI:** 10.1038/s41420-021-00707-6

**Published:** 2021-10-23

**Authors:** Xiulian Miao, Yan Guo, Sheng Zeng, Xingyu Liu, Xiao Teng, Luyang Li, Wenxuan Hong

**Affiliations:** 1 College of Life Sciences and Institute of Biomedical Research, Liaocheng University, Liaocheng, China; 2 Stem Cell Center, Affiliated Drum Tower Hospital of Nanjing University Medical School, Nanjing, China; 3 Department of Oral Medicine, The Affiliated Jiangning Hospital of Nanjing Medical University, Nanjing, China; 4 Institute of Biomedical Sciences, Fudan University, Shanghai, China

**Keywords:** Transcriptional regulatory elements, Epigenetics, Non-alcoholic fatty liver disease

## Abstract

Non-alcoholic fatty liver disease (NAFLD) is prototypical form of metabolic syndrome and has become a global pandemic. Hepatocytes undergo apoptosis in the pathogenesis of NAFLD. We report that the lymphokine LIGHT/TNFSF14 was upregulated in the murine NAFLD livers and in hepatocytes treated with free fatty acids (palmitate, PA). LIGHT knockdown or neutralization attenuated PA-induced apoptosis of hepatocytes. Similarly, knockdown or blockade of LTβR, the receptor for LIGHT, ameliorated apoptosis in hepatocytes exposed to PA. Ingenuity pathway analysis (IPA) revealed several Notch-related transcription factors as upstream regulators of LIGHT, of which HES5 expression was downregulated paralleling LIGHT induction in the pathogenesis of NAFLD. HES5 knockdown enhanced whereas HES5 over-expression weakened LIGHT induction in hepatocytes. HES5 was found to directly bind to the LIGHT promoter and repress LIGHT transcription. Mechanistically, HES5 interacted with SIRT1 to deacetylate histone H3/H4 on the LIGHT promoter to repress LIGHT transcription. SIRT1 knockdown or inhibition offset the effect of HES5 over-expression on LIGHT transcription and hepatocyte apoptosis. In conclusion, our data unveil a novel mechanism that might contribute to excessive apoptosis in hepatocyte exposed to free fatty acids.

## Introduction

Non-alcoholic fatty liver disease (NAFLD) is a prototypical metabolic disorder influenced by genetic and environmental factors. Well-established risk factors for NAFLD include obesity, type 2 diabetes, hypertension, hyperlipidemia, and senility [1]. NAFLD encompasses a continuum of pathologies ranging from simple steatosis, to steatohepatitis, to cirrhosis and hepatocellular carcinoma (HCC) [2]. NAFLD is projected to become the leading cause for HCC and liver transplantation causing significant socioeconomic burdens worldwide [3]. The pathogenesis of NAFLD is complex and remains incompletely understood despite decades of vigorous research. It is generally agreed that a combination of excessive influx and insufficient consumption of nutrients leads to skewed hepatic metabolism causing accumulation of lipid droplets in the liver. In response to lipotoxic stimuli, stressed hepatocytes may become necrotic and/or apoptotic, which triggers pro-inflammatory responses in the liver accelerating the pathogenesis of NAFLD [4]. Indeed, increased incidents of hepatocyte apoptosis have been observed in patients with NAFLD [5, 6]. On the contrary, manipulating key mediators of apoptosis, including caspase-3 [7] and caspase-6 [8], affects the development and progression of NAFLD in mice. In addition, a pan-caspase inhibitor (VX-166) has been reported to confer hepatoprotective effects on mice with established NAFLD [9].

Tumor necrosis factor superfamily member 14 (TNFSF14), also known as LIGHT, is a pro-apoptotic cytokine originally identified in activated T lymphocytes (hence the term “lymphokine”) and characterized as a mediator of host defense against the invasion of herpesvirus [10]. LIGHT has been reported to induce a pro-apoptotic response in a wide range of cells including thymocytes [11], islet beta cells [12], breast cancer cells [13, 14], osteoblasts [15], and smooth muscle cells [16] under both physiological and pathological conditions. Early investigation has found that LIGHT mediates cellular apoptosis by binding to lymphotoxin receptor beta (LTβR) [17]. Recent studies have implicated LIGHT in the pathogenesis of NAFLD. Otterdal et al. have reported that serum LIGHT levels are upregulated in the NAFLD patients and positively correlated with disease severity [18]. Herrero-Cervera et al. [19] have investigated the effect of LIGHT deletion on the pathogenesis of NAFLD in mice and found that LIGHT deficiency attenuated hepatic inflammation owing to defective leukocyte infiltration in a high-fat diet (HFD) induced model. These intriguing findings notwithstanding, it remains unclear how LIGHT expression is regulated and whether LIGHT contributes to hepatocyte apoptosis in the course of NAFLD pathogenesis. We report here that downregulation of transcriptional repressor HES5 leads to LIGHT upregulation and may contribute to hepatocyte apoptosis in the context of NAFLD.

## Results

### LIGHT expression is elevated in the pathogenesis of non-alcoholic fatty liver disease

**Fig. 1:**
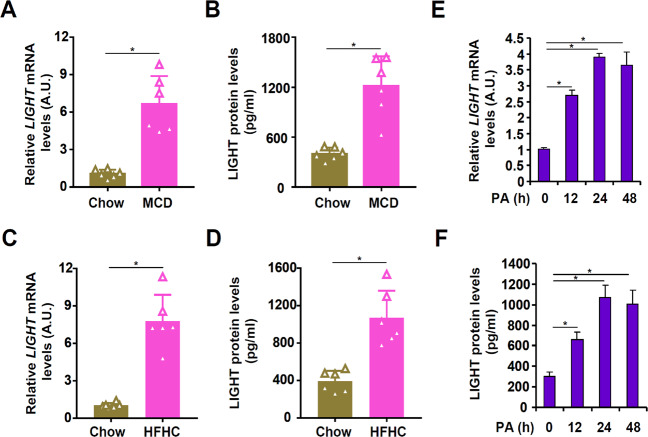
LIGHT expression is elevated in the pathogenesis of non-alcoholic fatty liver disease. **A**, **B** C57B6/L mice were fed an MCD diet for 4 weeks. Hepatic LIGHT expression was examined by qPCR and ELISA. **C**, **D** C57B6/L mice were fed an HFHC diet for 12 weeks. Hepatic LIGHT expression was examined by qPCR and ELISA. **E**, **F** Primary murine hepatocytes were treated with palmitate (0.3 mM) and harvested at indicated time points. LIGHT expression was examined by qPCR and ELISA.

In the first set of experiments, LIGHT expression was evaluated in different models of non-alcoholic fatty liver disease. Six to 8-week-old, male C57B6/L mice were fed a methionine-and-choline-deficient (MCD) diet for 4 weeks when extensive hepatocyte apoptosis was observed [20]. Compared to the mice fed on a control diet, the MCD diet-fed mice exhibited significantly higher LIGHT expression, as measured by qPCR and ELISA, in the livers (Fig. 1A, B). In the second model, 6–8-week-old, male C57B6/L mice were fed a high-fat high-carbohydrate diet (HFHC) for 12 weeks, which has been reported to induce hepatocyte apoptosis [21]. Significantly higher LIGHT expression, at both mRNA level (Fig. 1C) and protein level (Fig. 1D), was detected in the HFHC livers than in the control livers. Next, primary murine hepatocytes were exposed to free fatty acids (palmitate), a known risk factor for NAFLD [22] and inducer of hepatocyte apoptosis [23]. As shown in Fig. 1E, F, PA treatment led to robust induction of LIGHT expression in hepatocytes with the peak occurring at 24 h.

### LIGHT blockade might attenuate PA-induced hepatocyte apoptosis

**Fig. 2:**
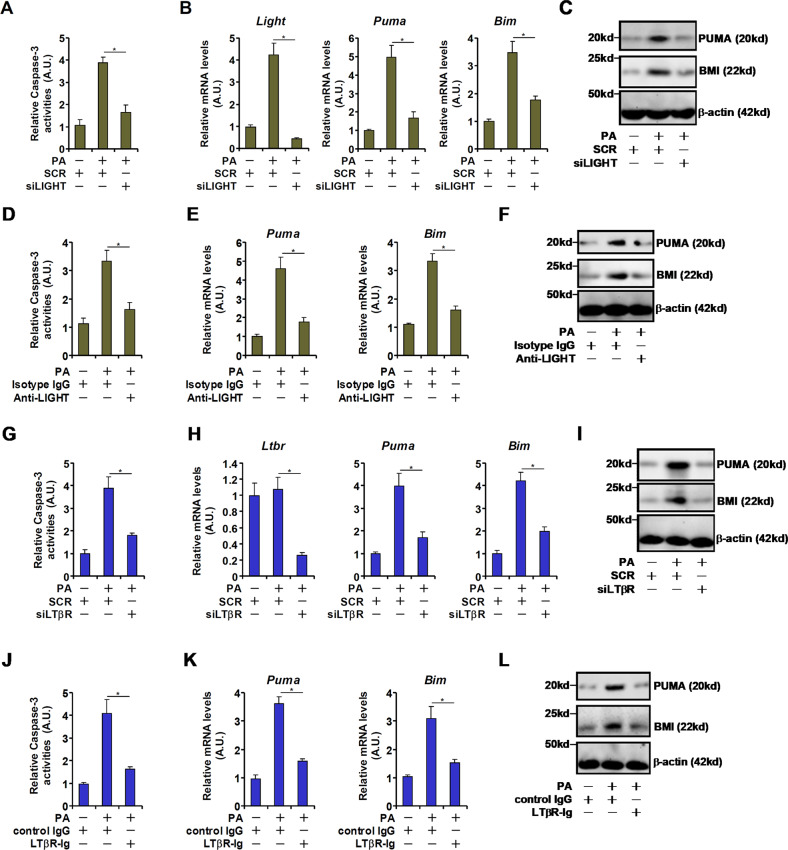
LIGHT blockade attenuates PA-induced hepatocyte apoptosis. **A**–**C** Primary murine hepatocytes were transfected with siRNA-targeting LIGHT or scrambled siRNA (SCR) followed by treatment with PA (0.3 mM). Caspase-3 activity was measured by a fluorescence kit as described in Methods. Gene expression was evaluated by qPCR and western blotting. **D**–**F** Primary hepatocytes were treated with PA (0.3 mM) in the presence or absence of a LIGHT neutralizing antibody. Caspase-3 activity was measured by a fluorescence kit as described in Methods. Gene expression was evaluated by qPCR and western blotting. **G**–**I** Primary murine hepatocytes were transfected with siRNA-targeting LTβR or scrambled siRNA (SCR) followed by treatment with PA (0.3 mM). Caspase-3 activity was measured by a fluorescence kit as described in Methods. Gene expression was evaluated by qPCR and western blotting. **J**–**L** Primary hepatocytes were treated with PA (0.3 mM) in the presence or absence of a LIGHT neutralizing antibody. Caspase-3 activity was measured by a fluorescence kit as described in Methods. Gene expression was evaluated by qPCR and western blotting.

Having observed elevated LIGHT expression in the pathogenesis of NAFLD, we asked whether LIGHT might contribute to hepatocyte apoptosis in this process. Several different strategies were exploited to interfere with LIGHT expression/signaling. A spectrophotometric assay, based on the detection of chromophore released by Caspase-3 catalyzed hydrolysis of its substrate, was employed to measure Caspase-3 activity. PA treatment led to increased apoptosis of primary murine hepatocytes as evaluated by Caspase-3 activity (Fig. 2A). In addition, mRNA (Fig. 2B) and protein (Fig. 2C) expression levels of pro-apoptotic genes were also upregulated by PA treatment. LIGHT knockdown by siRNA markedly attenuated PA-induced apoptosis of hepatocytes. Next, an anti-LIGHT neutralizing antibody was added to the culture media to prevent LIGHT from binding to its receptor [24]. Compared to the isotype IgG control, the LIGHT neutralizing antibody mitigated PA-induced apoptosis of hepatocytes (Fig. 2D–F). It is generally agreed that LIGHT exerts its pathobiological functions by binding to the trans-membrane receptor LTβR [25]. Indeed, depletion of endogenous LTβR with RNAi similarly ameliorated PA-induced apoptosis of hepatocytes (Fig. 2G–I). Finally, an LTβR antagonist (LTβR-Ig) [26] was added to the culture media along with PA. Blockade of the LIGHT-LTβR interaction and presumably the downstream signaling cascade suppressed PA-induced apoptosis of hepatocytes (Fig. 2J–L).

### HES5 downregulation parallels LIGHT upregulation in the pathogenesis of non-alcoholic fatty liver disease

**Fig. 3:**
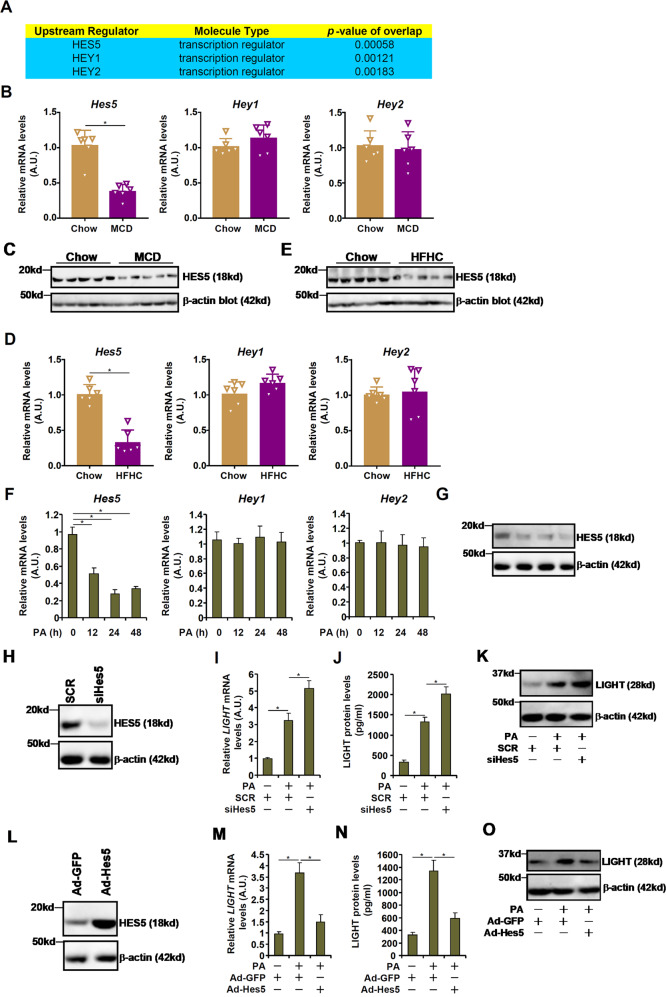
HES5 downregulation parallels LIGHT upregulation in the pathogenesis of non-alcoholic fatty liver disease. **A** IPA analysis of potential LIGHT upstream regulators. **B**, **C** C57B6/L mice were fed an MCD diet for 4 weeks. Hepatic gene expression was examined by qPCR and western. **D**, **E** C57B6/L mice were fed an HFHC diet for 12 weeks. Hepatic gene expression was examined by qPCR and western. **F**, **G** Primary murine hepatocytes were treated with palmitate (0.3 mM) and harvested at indicated time points. Gene expression was examined by qPCR and ELISA. **H**–**K** Primary murine hepatocytes were transfected with siRNA-targeting LIGHT or scrambled siRNA (SCR) followed by treatment with PA (0.3 mM). LIGHT expression was examined by qPCR, ELISA, and western blotting. **L**–**O** Primary murine hepatocytes were transduced with Ad-HES5 or Ad-GFP followed by treatment with PA (0.3 mM). LIGHT expression was examined by qPCR, ELISA, and western blotting.

As LIGHT expression was upregulated in the pathogenesis of NAFLD, the following experiments were performed to examine the potential mechanism(s). Ingenuity pathway analysis (IPA) revealed that several E-box-binding HEY basic helix-loop-helix (bHLH) transcription factors, considered to be mediators of the Notch signaling pathway [27], were among the top upstream regulators of LIGHT (Fig. 3A). Quantitative PCR (Fig. 3B, D) and western blotting (Fig. 3C, E) profiling indicated that HES5, but neither HEY1 nor HEY2, was downregulated in the livers of the NAFLD mice compared to the control mice in the MCD model and the HFD model. Similarly, PA treatment decreased the expression of HES5, but not that of HEY1 or HEY2, in primary murine hepatocytes (Fig. 3F, G). In addition, knockdown of HES5 (Fig. 3H) further augmented the induction of LIGHT expression by PA treatment in hepatocytes (Fig. 3I, J, K). On the contrary, over-expression of HES5, mediated by adenoviral delivery of a HES5 vector into hepatocytes (Fig. 3L), repressed LIGHT induction by PA treatment (Fig. 3M, N, O). Taken together, these data suggest that loss of HES5 expression may contribute to LIGHT upregulation in hepatocytes.

### HES5 directly binds to the LIGHT promoter and represses LIGHT transcription

**Fig. 4:**
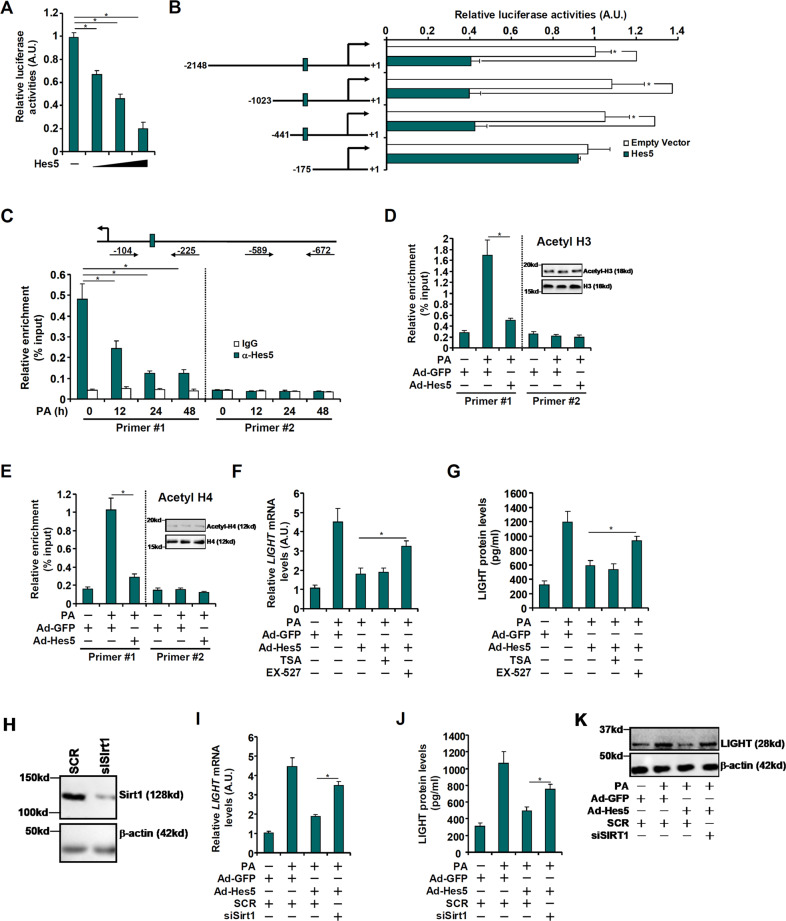
HES5 directly binds to the LIGHT promoter and represses LIGHT transcription. **A** A LIGHT promoter-luciferase construct (−2186/+1) were transfected into HepG2 cells with or without HEY5. Luciferase activities were normalized by protein concentration and GFP fluorescence. **B** Different LIGHT promoter-luciferase constructs were transfected into HepG2 cells with or without HES5. Luciferase activities were normalized by protein concentration and GFP fluorescence. **C** Primary murine hepatocytes were treated with palmitate (0.3 mM) and harvested at indicated time points. ChIP assay was performed with anti-HES5 or IgG. **D**, **E** Primary murine hepatocytes were transduced with Ad-HES5 or Ad-GFP followed by treatment with PA (0.3 mM). ChIP assay was performed with anti-acetyl H3 and anti-acetyl H4. Inset, global histone H3/H4 and acetyl H3/H4 levels were examined by western blotting. **F**, **G** Primary murine hepatocytes were transduced with Ad-HES5 or Ad-GFP followed by treatment with PA (0.3 mM) in the presence of absence of TSA (100 nM) or EX-527 (1 μM). LIGHT expression was examined by qPCR and ELISA. **H–K** Primary murine hepatocytes were transduced with Ad-HES5 or Ad-GFP, transfected with siRNA-targeting SIRT1 or scrambled siRNA (SCR) and treated with PA (0.3 mM). SIRT1 knockdown efficiency was examined by western. LIGHT expression was examined by qPCR, ELISA, and western blotting.

Since HES5 appeared to be able to regulate LIGHT expression in hepatocytes, we asked whether the regulation occurred at the transcriptional level. A LIGHT promoter-luciferase fusion construct (−2148/+1) was transfected into HepG2 cells with or without HES5. As shown in Fig. 4A, HES5 over-expression repressed the LIGHT promoter activity in a dose-dependent manner. To locate the HES5 binding element in the LIGHT promoter, serial inward deletions were introduced to the full-length construct. When the deletion was extended beyond −441 relative to the transcription start site, HES5 was no longer able to repress the LIGHT promoter (Fig. 4B). Closer examination of the LIGHT promoter region between −441 and −175 revealed a putative E-box (CACGTG) that could serve as a binding site for HES5. Chromatin immunoprecipitation (ChIP) assay showed that HES5 occupancy on the E-box of the LIGHT promoter was progressively decreased in response to PA treatment, mirroring the changes of HES5 expression (Fig. 4C). Of interest, PA treatment markedly induced the accumulation of acetylated histones H3 (Fig. 4D) and H4 (Fig. 4E) on the proximal, but not the distal, LIGHT promoter; HES5 over-expression suppressed the enrichment of both acetyl H3 and acetyl H4, indicating that HES5 might regulate LIGHT transcription by influencing histone (de)acetylation. To determine the specific histone deacetylase(s) mediating HES5-dependnet LIGHT trans-repression, hepatocytes were treated with either trichostatin A (TSA), a pan-inhibitor for class I/II deacetylases, or EX-527, a specific inhibitor for the class III deacetylase SIRT1. As shown in Fig. 4F, G, co-treatment with EX-527, but not TSA, partially reversed the repression of LIGHT expression by HES5 over-expression. Consistent with this observation, SIRT1 knockdown (Fig. 4H) also mitigated LIGHT repression by HES5 (Fig. 4I, J, K).

### HES5 interacts with SIRT1 to repress LIGHT transcription and antagonizes PA-induced apoptosis

**Fig. 5:**
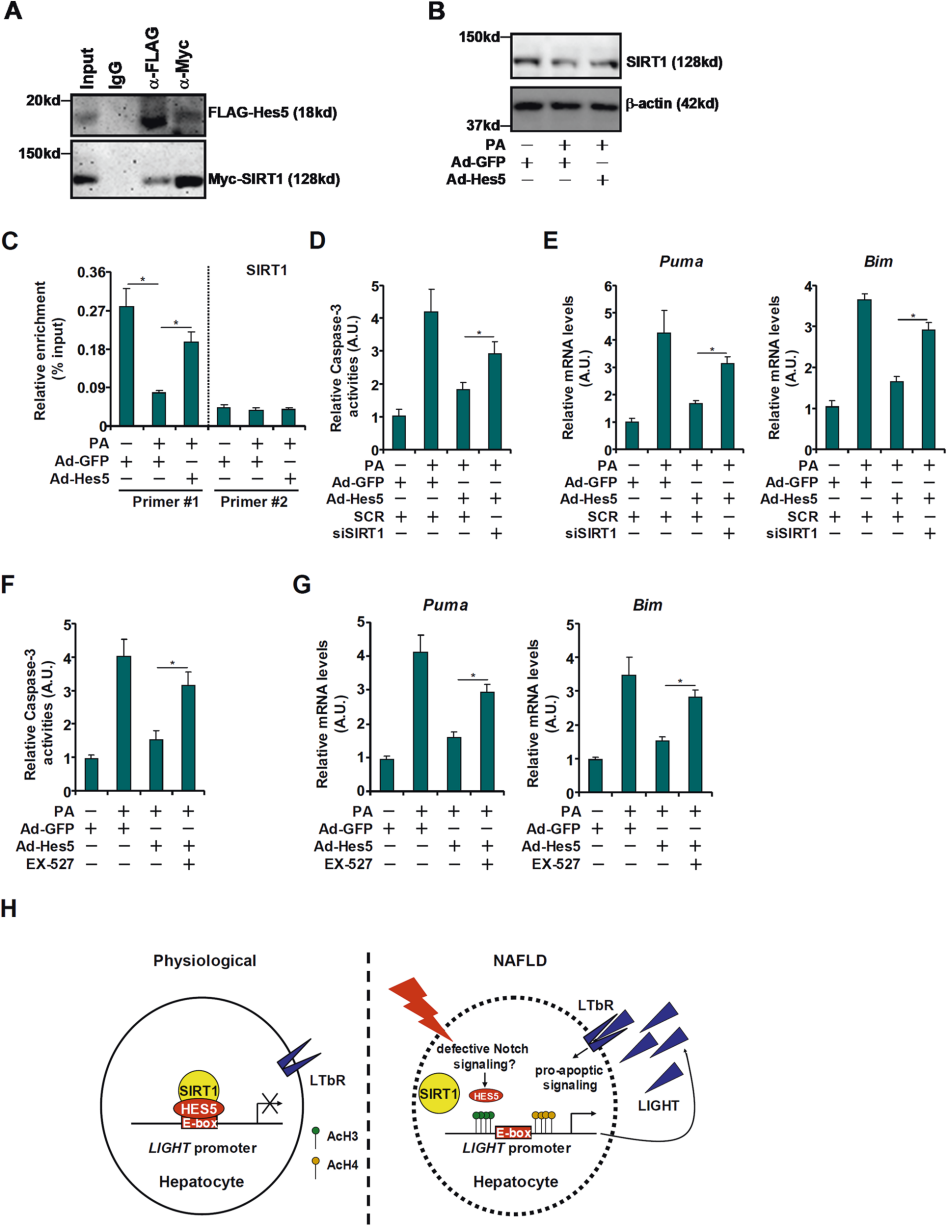
HES5 interacts with SIRT1 to repress LIGHT transcription and antagonizes PA-induced apoptosis. **A** FLAG-tagged HES5 and Myc-tagged SIRT1 were co-transfected into HEK293 cells followed by immunoprecipitation with anti-FLAG, anti-MYC, or IgG. **B**, **C** Primary murine hepatocytes were transduced with Ad-HES5 or Ad-GFP followed by treatment with PA (0.3 mM). SIRT1 expression was examined by western. SIRT1 binding to the LIGHT promoter was examined by ChIP. **D**, **E** Primary murine hepatocytes were transduced with Ad-HES5 or Ad-GFP, transfected with siRNA-targeting SIRT1 or scrambled siRNA (SCR) and treated with PA (0.3 mM). Caspase-3 activity was measured by a fluorescence kit as described in Methods. Gene expression was evaluated by qPCR. **F**, **G** Primary murine hepatocytes were transduced with Ad-HES5 or Ad-GFP followed by treatment with PA (0.3 mM) in the presence or absence of EX-527 (1 μM). Caspase-3 activity was measured by a fluorescence kit as described in Methods. Gene expression was evaluated by qPCR. **H** A schematic model.

In order to probe the possibility that HES5 may recruit SIRT1 to repress LIGHT transcription, the following experiments were performed. FLAG-tagged HES5 and Myc-tagged SIRT1 were co-transfected into HEK293 cells. Immunoprecipitation experiments demonstrated that an anti-FLAG antibody pulled down both HES5 and SIRT1 whereas an anti-Myc antibody precipitated both SIRT1 and HES5, suggesting that HES5 and SIRT1 could interact with each other (Fig. 5A). Neither PA treatment nor HES5 over-expression appeared to significantly alter SIRT1 expression hepatocytes (Fig. 5B). However, PA treatment downregulated SIRT1 recruitment to the proximal LIGHT promoter, which was restored by HES5 over-expression (Fig. 5C).

We finally evaluated the functional interplay between HES5 and SIRT1 in PA-induced hepatocyte apoptosis. Over-expression of HES5 antagonized PA-induced hepatocyte apoptosis as evidenced by caspase-3 activity (Fig. 5D) and expression levels of pro-apoptotic genes (Fig. 5E); simultaneous SIRT1 knockdown, however, abrogated the anti-apoptotic effect of HES5 over-expression. Similarly, SIRT1 inhibition by EX-527 treatment enabled apoptosis despite HES5 over-expression in PA-treated hepatocytes (Fig. 5F, G). Combined, these data suggest that HES5 may recruit SIRT1 to repress LIGHT transcription and antagonize hepatocyte apoptosis.

## Discussion

Lipotoxicity-associated apoptosis of hepatocytes is a hallmark event in the pathogenesis of NAFLD [28]. Here we describe a novel transcriptional mechanism that might contribute to free fatty acids (PA) induced hepatocyte apoptosis (Fig. 5H). We show that LIGHT/TNFSF14 expression is upregulated by pro-NAFLD stimuli in the murine livers and by PA treatment in primary murine hepatocytes. Although LIGHT was originally isolated from and characterized in T lymphocytes [10], recent studies suggest that its expression could be detected, at least under stress conditions, in a variety of different cell types. Kim et al. have observed that white adipocytes exposed to PA treatment upregulate the production and release of LIGHT, which serves as a chemoattractant to promote immune cell infiltration [29]. Grabiec et al. [30] have reported that PA treatment induces LIGHT expression by more than threefold in proliferating skeletal muscle cells, which likely accounts for reduced viability of the myocytes. In agreement with our data, Saunders et al. [31] have demonstrated that LIGHT expression is upregulated in the murine livers by high-fat diet (HFD) feeding. Despite these consistent observations, the potential effect of LIGHT and its receptor LTβR on the full spectrum of NAFLD pathogenesis has not been clearly and conclusively elucidated. Heikenwalder and colleagues have shown that global LIGHT deletion or hepatocyte-restricted LTβR deletion in mice attenuates the development of HCC in mice following 12-month feeding on a choline-deficient high-fat diet (CD-HFD) [32]. Of interest, LIGHT deficiency attenuates liver injuries as assessed by plasma ALT levels, which could be owing to reduced hepatocyte apoptosis although it was not directly evaluated. On the contrary, the LIGHT^-/-^ mice placed on a 42% high-fat diet for 12 weeks displayed exacerbated obesity and insulin resistance, two key features of NAFLD, compared to the wild type mice [31]. As WT mice receiving bone marrows from the LIGHT^-/-^ mice phenocopy the LIGHT^-/-^ mice, it is proposed that hematopoietic cells likely mediate the pro-NAFLD effects of LIGHT deficiency. More recently, Herrero-Cervera et al. have fed the LIGHT^-/-^ mice with a high-fat high-cholesterol diet (HFHCD) for 16 weeks and discovered that LIGHT inactivation improved glucose tolerance and insulin sensitivity [19]. These discrepancies likely reflect the differences in the diet-induced models. Alternatively, LIGHT in different cell lineages may exert distinct or opposing effects on the pathogenesis of NAFLD.

We show here that LIGHT upregulation in hepatocytes can be attributed to, at least in part, by loss of HES5 expression. HES5 can be placed downstream of the Notch signaling pathway [33]. The mechanism whereby HES5 is downregulated in the liver by pro-NAFLD stimuli is not clear. A recent population study by Auguet et al. has found an inverse correlation between hepatic Notch signaling and NAFLD severity in women with obesity [34], which likely explains HES5 downregulation in the pathogenesis of NAFLD. HES5-null mice are viable but with some minor neurological abnormalities [35, 36]. Although it remains to be determined how HES5 deficiency would influence the pathogenesis of NAFLD in mice, two recent reports appear to support a role for HES5 in the regulation of liver pathobiology. Yu et al. have investigated the involvement of HES5 in hepatic ischemia-reperfusion injury (IRI) and found that HES5, activated by canonical Notch signaling, protects the mice from IRI by cleansing excessive reactive oxygen species in hepatocytes [37]. On the other hand, Luiken et al. have found that high HES5 expression predicts better survival in a small cohort of patients with hepatocellular carcinoma and that HES5 over-expression suppresses MYC-induced hepato-carcinogenesis in mice [38]. As ROS accumulation represents a key pathophysiological process and a driving force in NAFLD and the development of HCC is the ultimate consequence of NAFLD, it is tempting to speculate that enhancing HES5 activity may attenuate NAFLD pathogenesis in vivo.

HES5 typically interacts with histone deacetylases to repress target gene transcription [27]. Here, we show that HES5 recruits the class III lysine deacetylase SIRT1 and cooperates with SIRT1 to repress LIGHT transcription in hepatocytes. SIRT1 has long been hailed as a master regulator of hepatic metabolism with a well-established protective role in NAFLD [39]. Of interest, diminished SIRT1 expression/activity in the NAFLD livers has been found to be associated with increased hepatocyte apoptosis in mice and in humans [40, 41]. Notably, SIRT1 may contribute to the regulation of hepatocyte apoptosis in NAFLD via multiple mechanisms. For instance, SIRT1 has been shown to deacetylate and consequently activate NF-κB, which in turn promotes the transcription of several anti-apoptotic genes in hepatocytes [42]. Alternatively, SIRT1 may deacetylate and de-activate FOXO3a thereby excluding FOXO3a from the nucleus and preventing FOXO3a from stimulating the transcription of pro-apoptotic genes [43]. Our data offer additional support for SIRT1 as a hepato-protector by reining in excessive loss of hepatocytes due to lipotoxicity-induced apoptosis.

In conclusion, our data as summarized in this report point to a HES5-SIRT1-LIGHT axis that can potentially regulate hepatocyte apoptosis in NAFLD pathogenesis. There are lingering issues that need to be addressed in future studies. For instance, although previous investigations have indicated that LIGHT is capable of modulating both the intrinsic and extrinsic apoptotic pathways [14, 44, 45], it is not clear how LIGHT regulates apoptosis of hepatocytes in the context of NAFLD. Several HES5 targeting reagents, including neutralizing antibody and receptor antagonists, are currently available and proven effective in the intervention of glomerulonephritis [46] and Sjögren’s syndrome [47]. Our data certainly provide new incentive to exploit these reagents as potential therapeutic solutions against NAFLD.

## Methods

### Animals

All animal protocols were reviewed and approved the intramural Ethics Committee on Humane Treatment of Laboratory Animals of Nanjing Medical University. The mice were maintained in an SPF environment with 12 h light/dark cycles and libitum access to food and water. Non-alcoholic fatty liver disease (NAFLD) was induced by MCD feeding or HFHC feeding as previously described [48, 49].

### Cell culture

Primary hepatocytes were isolated from C57B6/L mice and cultured in DMEM with 10% FBS as previously described [48, 50–52]. FLAG-tagged Hes5 [53], LIGHT promoter-luciferase constructs [54], and MYC-tagged SIRT1 [55] have been described previously. Small interfering RNAs were purchased from Dharmacon. Transient transfections were performed with Lipofectamine LTX (for DNA plasmid) or Lipofectamine RNAiMax (For siRNA) per vendor recommendation. Luciferase activities were assayed 24-48 hours after transfection using a luciferase reporter assay system (Promega) as previously described [56–58].

### Whole-cell lysate extraction, immunoprecipitation, and western blotting

Whole-cell lysates were extracted by re-suspending the cell pellet in RIPA buffer (50 mM Tris pH 7.4, 150 mM NaCl, 1% NP40, 0.5% sodium deoxycholate, 0.1% SDS) with freshly added protease inhibitor tablet (Thermo Fisher) as previously described [59–66]. Specific antibodies or pre-immune IgGs (P.I.I.) were added to and incubated with cell lysates overnight before being absorbed by Protein A/G-plus Agarose beads (Santa Cruz). Precipitated immune complex was released by boiling with 1x SDS electrophoresis sample buffer. Thirty micrograms of protein were loaded in each lane and separated by 8% PAGE-SDS gel with all-blue protein markers (Bio-Rad). Proteins were transferred to nitrocellulose membranes (Bio-Rad) in a Mini-Trans-Blot Cell (Bio-Rad). The membranes were blocked with 5% fat-free milk powder in Tris-buffered saline at room temperature for half an hour and then incubated with anti-Hes5 (Abcam, ab194111), anti-SIRT1 (Santa Cruz, sc-74504), anti-FLAG (Sigma, F3165), anti-MYC (Thermo Fisher, PA1-981), anti-LIGHT (Thermo Fisher, PA5-104479), anti-PUMA (Proteintech, 55120-1), anti-BIM (Proteintech, 22037-1), and anti-β-actin (Sigma, A1978) overnight. Image J software was used for densitometrical quantification and densities of target proteins were normalized to those of β-actin. Data are expressed as relative protein levels compared to the control group, which is arbitrarily set as 1.

### RNA isolation and real-time PCR

RNA was extracted with the RNeasy RNA isolation kit (Qiagen). Reverse transcriptase reactions were performed using a SuperScript First-strand Synthesis System (Invitrogen) as previously described [62, 67–72]. Real-time PCR reactions were performed on an ABI Prism 7500 system with the following primers: *Light*, 5’-GTTTCTCCTGAGACTGCATCAA-3’ and 5’-TGGCTCCTGTAAGATGTGCTG-3’; *Hes5*, 5’-AGTCCCAAGGAGAAAAACCGA-3’ and 5’-GCTGTGTTTCAGGTAGCTGAC-3’; *Hey1*, 5’-GCGCGGACGAGAATGGAAA-3’ and 5’-TCAGGTGATCCACAGTCATCTG-3’; *Hey2*, 5’-AAGCGCCCTTGTGAGGAAAC-3’ and 5’-GGTAGTTGTCGGTGAATTGGAC-3’; *Puma*, 5’-AGCAGCACTTAGAGTCGCC-3’ and 5’-CCTGGGTAAGGGGAGGAGT-3’; *Bim*, 5’-TCGTCCATCGAGGATGACTTC-3’ and 5’-TGCAGAGAGAGGATACTGTAGAC-3’. Ct values of target genes were normalized to the Ct values of housekeeping control gene (18s, 5’-CGCGGTTCTATTTTGTTGGT-3’ and 5’-TCGTCTTCGAAACTCCGACT-3’) using the ΔΔCt method and expressed as relative mRNA expression levels compared to the control group, which is arbitrarily set as 1.

### Chromatin immunoprecipitation (ChIP)

Chromatin immunoprecipitation (ChIP) assays were performed essentially as described before [57, 73–90]. In brief, chromatin in control and treated cells were cross-linked with 1% formaldehyde. Cells were incubated in lysis buffer (150 mM NaCl, 25 mM Tris pH 7.5, 1% Triton X-100, 0.1% SDS, 0.5% deoxycholate) supplemented with protease inhibitor tablet and PMSF. DNA was fragmented into ~200 bp pieces using a Branson 250 sonicator. Aliquots of lysates containing 200 μg of protein were used for each immunoprecipitation reaction with anti-Hes5 (Abcam, ab194111), anti-SIRT1 (Santa Cruz, sc-74504), anti-acetyl H3 (Millipore, 06-599), anti-acetyl H4 (Millipore, 06-866), or pre-immune IgG. Precipitated DNA was amplified with the following primers: #1, 5’-AGAGTGAGACAGGGCCAAGAC-3’ and 5’-AAACCGAAATTGCTCAACACAC-3’; #2, 5’-AAACCCACAACGTATTA-3’ and 5’-ATGCAGCAATGAACAAC-3’.

### Enzyme-linked immunosorbent assay

Secreted LIGHT levels were examined by ELISA as previously described using a commercially available kit (R&D, catalog# DY1794-05) according to vendor’s recommendations.

### Fluorometric caspase-3 activity assay

Caspase-3 activity in cell lysates was assayed by a fluorometric kit using a microtiter plate reader per vendor instructions (Abcam, ab39401). Briefly, chromophore p-nitroaniline (p-NA) is conjugated to the Caspase-3 substrate DEVD. When incubated with cell lysates containing activate Caspase-3, p-NA is cleaved from DEVD and released. The light emission is measured at 400 or 405 nm on a a GloMax microplate reader (GM3000, Promega). The data were expressed as relative Caspase-3 activity compared to the control group arbitrarily set as 1.

### Statistical analysis

Two-tailed student *t*-test or one-way ANOVA with post hoc Scheff´e analyses was performed by SPSS software (IBM SPSS v18.0, Chicago, IL, USA). Unless otherwise specified, values of *p* < 0.05 were considered statistically significant.

## Supplementary information

authorship change consents

## Data Availability

The data that support the findings of this study are available upon reasonable request.
