# Supplementary material for: HES5-mediated repression of LIGHT transcription may contribute to apoptosis in hepatocytes
Source: Cell Death Discov. 2021 Oct 23;7:308. doi: 10.1038/s41420-021-00707-6 (PMC8542050; doi:10.1038/s41420-021-00707-6)
Supplement: Supplementary file 1 — authorship change consents [file 41420_2021_707_MOESM1_ESM.pdf]

Wenxuan Hong
Wed 10/6/2021 2:51 PM
To: You

I consent.

...

From: Wenxuan Hong <wxhongibs@hotmail.com>
Sent: Wednesday, October 6, 2021 2:46 PM
To: lylinjmu@hotmail.com <lylinjmu@hotmail.com>; szengdrumtower@hotmail.com <szengdrumtower@hotmail.com>; xlmiaolcu@hotmail.com <xlmiaolcu@hotmail.com>
Cc: Wenxuan Hong <wxhongibs@hotmail.com>
Subject: authorship change

This is to inform all the authors that Miss Xiao Teng has been added as a co-author to the manuscript "HES5-mediated repression of LIGHT transcription may contribute to apoptosis in hepatocytes" currently under consideration by Cell Death Discovery. Please send your consent by replying directly to this email. Thank you. WX

Sheng Zeng <szengdrumtower@hotmail.com>
Wed 10/6/2021 2:54 PM
To: You

I consent.

...

From: Wenxuan Hong <wxhongibs@hotmail.com>
Sent: Wednesday, October 6, 2021 2:46 PM
To: lylinjmu@hotmail.com <lylinjmu@hotmail.com>; szengdrumtower@hotmail.com <szengdrumtower@hotmail.com>; xlmiaolcu@hotmail.com <xlmiaolcu@hotmail.com>
Cc: Wenxuan Hong <wxhongibs@hotmail.com>
Subject: authorship change

This is to inform all the authors that Miss Xiao Teng has been added as a co-author to the manuscript "HES5-mediated repression of LIGHT transcription may contribute to apoptosis in hepatocytes" currently under consideration by Cell Death Discovery. Please send your consent by replying directly to this email. Thank you. WX

Xiulian Miao <xlmiaolcu@hotmail.com>
Wed 10/6/2021 3:01 PM
To: You

I consent.

...

From: Wenxuan Hong <wxhongibs@hotmail.com>
Sent: Wednesday, October 6, 2021 2:46 PM
To: lylinjmu@hotmail.com <lylinjmu@hotmail.com>; szengdrumtower@hotmail.com <szengdrumtower@hotmail.com>; xlmiaolcu@hotmail.com <xlmiaolcu@hotmail.com>
Cc: Wenxuan Hong <wxhongibs@hotmail.com>
Subject: authorship change

This is to inform all the authors that Miss Xiao Teng has been added as a co-author to the manuscript "HES5-mediated repression of LIGHT transcription may contribute to apoptosis in hepatocytes" currently under consideration by Cell Death Discovery. Please send your consent by replying directly to this email. Thank you. WX

Luyang Li <lylinjmu@hotmail.com>
Wed 10/6/2021 3:03 PM
To: You

I consent

...

From: Wenxuan Hong <wxhongibs@hotmail.com>
Sent: Wednesday, October 6, 2021 2:46 PM
To: lylinjmu@hotmail.com <lylinjmu@hotmail.com>; szengdrumtower@hotmail.com <szengdrumtower@hotmail.com>; xlmiaolcu@hotmail.com <xlmiaolcu@hotmail.com>
Cc: Wenxuan Hong <wxhongibs@hotmail.com>
Subject: authorship change

This is to inform all the authors that Miss Xiao Teng has been added as a co-author to the manuscript "HES5-mediated repression of LIGHT transcription may contribute to apoptosis in hepatocytes" currently under consideration by Cell Death Discovery. Please send your consent by replying directly to this email. Thank you. WX

Reply | Forward

Xingyu Liu <xyliulcu@outlook.com>
Wed 10/6/2021 3:11 PM
To: You

I consent.

...

From: Wenxuan Hong <wxhongibs@hotmail.com>
Sent: Wednesday, October 6, 2021 2:47 PM
To: yguolcu@hotmail.com <yguolcu@hotmail.com>; xyliulcu@outlook.com <xyliulcu@outlook.com>; txiaolcu@hotmail.com <txiaolcu@hotmail.com>
Subject: authorship change

This is to inform all the authors that Miss Xiao Teng has been added as a co-author to the manuscript "HES5-mediated repression of LIGHT transcription may contribute to apoptosis in hepatocytes" currently under consideration by Cell Death Discovery. Please send your consent by replying directly to this email. Thank you. WX

Yan Guo <yguolcu@hotmail.com>
Wed 10/6/2021 3:13 PM
To: You

I consent.

...

From: Wenxuan Hong <wxhongibs@hotmail.com>
Sent: Wednesday, October 6, 2021 2:47 PM
To: yguolcu@hotmail.com <yguolcu@hotmail.com>; xyliulcu@outlook.com <xyliulcu@outlook.com>; txiaolcu@hotmail.com <txiaolcu@hotmail.com>
Subject: authorship change

This is to inform all the authors that Miss Xiao Teng has been added as a co-author to the manuscript "HES5-mediated repression of LIGHT transcription may contribute to apoptosis in hepatocytes" currently under consideration by Cell Death Discovery. Please send your consent by replying directly to this email. Thank you. WX

Reply | Forward

TX

Teng Xiao <xtenglcu@hotmail.com>

Wed 10/6/2021 3:19 PM

To: You

I consent.

...

寄件者: Wenxuan Hong <wxhongibs@hotmail.com>
寄件日期: 2021年10月6日 下午 03:18
收件者: xtenglcu@hotmail.com <xtenglcu@hotmail.com>
主旨: authorship change

This is to inform all the authors that Miss Xiao Teng has been added as a co-author to the manuscript "HES5-mediated repression of LIGHT transcription may contribute to apoptosis in hepatocytes" currently under consideration by Cell Death Discovery. Please send your consent by replying directly to this email. Thank you. WX

Reply | Forward
